# Seasonal Differences in Determinants of Time Location Patterns in an Urban Population: A Large Population-Based Study in Korea

**DOI:** 10.3390/ijerph14070672

**Published:** 2017-06-22

**Authors:** Sewon Lee, Kiyoung Lee

**Affiliations:** Department of Environmental Health, Graduate School of Public Health, Seoul National University, 1 Gwanak-ro, Gwanak-gu, Seoul 88026, Korea; sewonv@snu.ac.kr

**Keywords:** exposure modeling, personal exposure, population based studies

## Abstract

Time location patterns are a significant factor for exposure assessment models of air pollutants. Factors associated with time location patterns in urban populations are typically due to high air pollution levels in urban areas. The objective of this study was to determine the seasonal differences in time location patterns in two urban cities. A Time Use Survey of Korean Statistics (KOSTAT) was conducted in the summer, fall, and winter of 2014. Time location data from Seoul and Busan were collected, together with demographic information obtained by diaries and questionnaires. Determinants of the time spent at each location were analyzed by multiple linear regression and the stepwise method. Seoul and Busan participants had similar time location profiles over the three seasons. The time spent at own home, other locations, workplace/school and during walk were similar over the three seasons in both the Seoul and Busan participants. The most significant time location pattern factors were employment status, age, gender, monthly income, and spouse. Season affected the time spent at the workplace/school and other locations in the Seoul participants, but not in the Busan participants. The seasons affected each time location pattern of the urban population slightly differently, but overall there were few differences.

## 1. Introduction

Time location data are important in estimating the level of exposure to air pollutants using exposure assessment models. The time spent at locations can be combined with micro-environmental pollutant concentrations for estimation of personal exposures. Examples of personal exposure estimate models using time location data are the probabilistic National Ambient Air Quality Standards Exposure Model (pNEM) and Air Pollution Exposure Distributions within Adult Urban Populations in Europe (EXPOLIS) [1,2]. Exposure models based on time location data are essential in assessment of pollutant exposure for risk assessment and epidemiologic studies [3].

Different countries use time location data differently. In 2004, time location pattern data from a nationally representative population was collected in Korea. During weekdays, the Korean population spent 59.3% and 7.3% of their time at home and on transportation, respectively [4]. When time location data were collected in 2009, the Seoul population spent 58.3% and 8.3% of their time during weekdays at home and on transportation, respectively [5]. Extensive population time location data has been collected by the Canadian Human Activity Pattern Survey (CHAPS, CHAPS2) in Canada, through the National Human Activity Pattern Survey (NHAPS) in the United States, and EXPOLIS in Europe in the 1990s [6,7,8,9]. Time spent at home approximated 69.9%, 68.7% and 56.2–65.7% in Canada, the US, and European cities, respectively. Different time location patterns suggested that local information is required for better exposure estimation in those areas.

Time location data can be affected by various factors. Region, weekday, and socio-demographic factors such as age, gender, ethnicity, income, education, and employment status have all been found to be significant factors in time location patterns [8,9,10,11,12]. Time location patterns differ by region [8]. On weekdays, the Korean population has been shown to spend less time at home and more time on transportation compared with weekends [4]. Adults spent more time in the workplace/school and spent less time at home compared with other age groups [10,11,13]. Females spent more time at home and less time on transportation than males [4,11]. Those who were Chinese, received low-income, and were highly educated spent more time at home than other ethnic groups [12].

Differences in time location patterns by season have not been clearly defined. Most studies have determined that there are differences in time location pattern between summer and winter [6,8,9,10,11]. Time spent at home tended to be longer in winter than in summer. A longitudinal study of one individual (one year) suggested seasonal impact on time spent indoors [14]. Several studies have explored time location data for various seasons. The seasonal effects on time location pattern varied among European urban cities [7]. A comparison of seasonal effect with the various seasons in urban populations is needed.

The purposes of the study were to present national survey on time location pattern in urban cities over three seasons and to compare the individual factors associated with time spent in various micro-environments over the three seasons. This national survey in Korea collected 32,427 person-days on weekdays and 21,549 person-days at weekends. This particular study analyzed the weekday data for the 32,427 person-days.

## 2. Materials and Methods

### 2.1. Time Use Survey

The source of the time location pattern data in 2014 was from Time Use Survey of Korean Statistics (KOSTAT). More information on the study participant selection method are published on the website (http://meta.narastat.kr/metasvc/index.do?ConfmNo=101052&amp;inputYear=2014). Briefly, in 2014, 269,664 administrative areas were arranged by classification indicators, which affected time use in 2005. Eight hundred administrative areas were systematically selected according to probability proportional to size method. A total of 12,000 households were extracted by simple random sampling. This equated to 15 households from each administrative area. Out of a possible 12,000 households 11,986 households participated in the survey. From 27,716 identified participants, a total of 26,988 subjects older than 10 years of age agreed to participate in this study. The survey was conducted in summer (18 July–27 July), fall (19 September–1 October) and winter (28 November–7 December) in 2014. Diary data for 32,427 person-days on weekdays and 21,549 person-days at weekends were collected in all areas in Korea.

Each subject completed a questionnaire concerning time location information on 10-min intervals for two consecutive days regardless of the day of week. Demographic, socioeconomic, and family information were surveyed. The locations were classified into nine micro-environments: own home, workplace/school, other home, restaurant/bar, other locations including outdoors, walk, personal transportation, public transportation, and other transportation.

### 2.2. Data Analysis

For analysis of time location pattern of urban population according across three seasons, weekday data were extracted from 3984 person-days in Seoul and 2171 person-days in Busan. Data on person-days were assumed to represent person data. Descriptive statistics were used to analyze the time spent in the nine places in the two cities. In each city, an ANOVA model was used to determine the difference in time spent in each location over three seasons, in each city. Determinants of the time spent at nine locations in two cities over the three seasons were analyzed by multiple linear regression and the stepwise method. Working status, age, gender, monthly income, health status, educational level, and season were independent variables in the analysis. All variables were dichotomized except age and monthly income which were continuous variables. The Statistical Package for the Social Sciences (SPSS) version 23 (IBM SPSS Statistics, Armonk, NY, USA) was used for all statistical analysis.

## 3. Results

### 3.1. Survey Participant Characteristics

Seoul and Busan participants were selected for time-activity patterns in urban populations. Seoul is the capital of Korea where 10,103,233 people reside. Busan is the second most populated city with 3,519,401 (Ministry of the Interior, 2015). KOSTAT data included 3981 and 2171 population data sets for Seoul and Busan, respectively. Gender and age characteristics of study participants were similar to those of the original population. The distribution of participants by age and gender with the original population are provided in Table 1.

### 3.2. Time Profiles of Study Participants at Various Locations in Two Cities by Season

**Figure 1:**
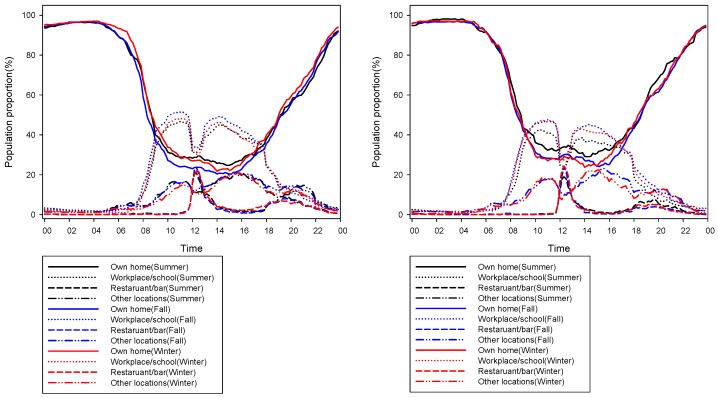
Percentage of Seoul (**Left**) and Busan (**Right**) participants in 4 locations by the time of day over three seasons.

Since the pollutants in locations varied temporarily, it was necessary to examine the percentage of the study populations for exposure assessment according to the time of day. The percentage of participants in each of the four varied locations was compared over three seasons, according to the time of day (Figure 1). Over the three seasons, more than 85% of participants stayed at home from 11:00 PM to 6:30 AM and this value was similar across the individual seasons. In the morning, more than 40% of participants stayed at the workplace/school. Approximately 20% of the participants visited a restaurant/bar at lunchtime. After 8:00 PM, the percentage of participants at workplace/school was less than 20% in both cities. The percentages of participants in each location according to time of day were similar over the three seasons in the two cities.

### 3.3. The Time Spent at Micro-Environments in Two Cities by Season

We considered participants who visited locations and the time spent at various locations in Seoul and Busan (Table 2 and Table 3). Over three seasons, there was no significant difference in the percentage of those who visited each location in both cities. The time spent at all locations were not significantly different for the two cities. With respect to Seoul participants, the time spent at locations was not different except for those who stayed within their own home (*p* < 0.01), walked (*p* < 0.01) or took other transportation (*p* = 0.038). Seoul participants stayed 867.7 ± 9.1, 843.7 ± 6.2, and 890.0 ± 8.3 min in their own home; spent 52.4 ± 1.2, 47.8 ± 0.9, and 45.3 ± 1.1 min walking; and 61.0 ± 4.9, 73.3 ± 4.3, and 88.8 ± 10.3 min on other transportation in summer, fall, and winter, respectively (Table 2). In Busan participants, the time spent at locations were not significantly different except for time spent in a restaurant/bar (*p* < 0.01). Busan participants stayed 74.9 ± 4.1, 59.9 ± 2.2, and 58.0 ± 2.6 min at the restaurant/bar in summer, fall and winter, respectively (Table 3).

### 3.4. Factors That Influence Time Location Pattern in Two Cities by Season

Table 4 and Table 5 show the effects of influential variables using multiple linear regression models on total time spent at 6 locations. Working conditions, age, gender, monthly income, spouse, health status, education, season, and agricultural status were factors entered into the regression model. Other variables for time spent at home, walk, and on other transportation were excluded in these two tables.

In Seoul, season affected the time spent in the workplace/school and at other locations. Monthly income also influenced the time spent at six locations. The time spent at five locations (except on public transportation) was affected by age and gender. Education affected the time spent at locations except in other locations. Working conditions and spouse affected the time spent at four locations. Health conditions affected the time spent at two locations. Agricultural status did not affect the time spent at any of the locations. The coefficients of determination were 0.424 for the time spent in own home and 0.448 for time spent at the workplace/school. The coefficients of determination were less than 0.13 for the time spent at other locations (Table 4).

In Busan, season did not affect the time spent at any location. Working status and gender influenced the time spent at five locations except for restaurant/bar and public transportation, respectively. Age, monthly income, education, and spouse affected the time at four locations. Health conditions and agricultural status influenced one and two of the six locations, respectively. The coefficients of determination were 0.456 for the time spent in own home, 0.460 for time spent at the workplace/school and 0.257 for private transportation. The coefficients of determination were less than 0.10 for the time spent in other locations (Table 5). The factors that influence the time spent at other home, walk, and on other transportation in Seoul and Busan are included in Tables S1 and S2.

## 4. Discussion

The time profile pattern of those study participants who spent most of their time at home during the 24-h day was similar to another Korean study [4]. Like the 2004 study, the majority of Seoul and Busan participants in our survey left their home in the morning and came home in the evening during the 24-h day. On weekdays, the difference in time spent at home between the Seoul population in 2004 and 2014 was less than 36 min. In Busan, the difference in time spent at home between populations in 2004 and 2014 was less than 79 min.

The time location pattern of the Korean population is different to that of other developed countries. Compared to a Canadian study, Korean participants in Seoul and Busan spent less time at home (58.6–61.8%, 62.4–64.8%) than Canadians did (69.9%) [11]. Indeed, Seoul participants spent less time at home than Americans adults in both summer (62.4%) and winter (67.4%) [10]. However, the time spent at the workplace/school of Seoul (32.0–33.3%) and Busan participants (31.3–32.0%) exceeded that of American (8.6–10.0%) and Canadian (8.6–10.0%) adults [10]. Furthermore, the participants in the two cities spent less time at home and more time at workplace/school than Minneapolis cohorts who spent 71% of their time at home and 13% of time at the workplace/school over the 24-h day [15]. On average, the time spent at home ranged from 61.1% in Grenoble (France) to 65.8% in Oxford (England). The average time at workplaces per day ranged from 25.4% in Athens (Greece) to 31.3% in Milan (Italy) and Prague (Czech Republic). Seoul and Busan study participants spent more time at home than residents in European counterparts from Helsinki, Basel, Milan, and Prague. The time spent in the workplace/school by Seoul and Busan participants exceeded that of populations from seven European cities [7]. According to Organization for Economic Co-operation and Development (OECD) statistics, Korean employees worked for 2113 h in 2015 on average, which was much higher than those of Canadian (1706 h), American (1790 h), and European employees (1371–1674 h) [16]. This could explain the observation here that the Korean population spends more time at the workplace/school and less time at home in comparison with Canadian, American, and European populations. Seoul and Busan study participants stayed longer at home than study participants from Hong Kong who spent 58% of their time at home during the 24-h day [17]. The time location patterns among Asian and Western countries might be varied due to cultural and societal difference such as working hours, school hours, and entertainment choices [4].

Working conditions, gender, spouse, monthly income, age, education, agricultural, and health conditions were the variables included in the multiple regression model in this study. Although health conditions may have influenced participant duration in certain places, it only affected those of two and one place in Seoul and Busan, respectively. In this study, all variables except health conditions were associated with similar time spent at different places to other American, Canadian, and Korean studies [4,8,10,12,13]. Region, weekday, and socio-demographic factors such as age, gender, ethnicity, income, education, and employment status have been identified as significant factors that can influence time location patterns. Time location patterns are different according to EPA regions [8]. The Korean population stays longer at home and spends less of their time on transportation on weekdays than at weekends [4]. Adults spent more time in the workplace/school and less time at home than other age groups [10,11,13]. Females spent more of their time at home and less time on transportation than males [4,11]. Those populations that were Chinese, highly educated, and received low-incomes stayed longer at home than other ethnicities [12].

Season had little impact on the time spent in various places in Seoul and Busan. Season influenced the time spent at workplace/school and other locations among nine locations of the Seoul population but this was not observed in the Busan population. This result is different compared with the time location pattern observed in other large populations [7,11,14]. In US Environmental Protection Agency’s Consolidated Human Activity Database (CHAD) study, the time spent at locations was different in specific seasonal pairs, and not for all seasons [14]. The seasonal effect in all European cities is limited because the specific relevance of seasonal effect with time location pattern is generally different among European cities [7]. The time spent at home and outdoors was different but time at other indoor locations and in vehicles was comparable for summer and winter in a Canadian study [11]. The time spent at home in Helsinki decreased as the weather got warmer from winter to spring, regardless of age. Furthermore, outdoors and transportation time increased, as it got warmer [18]. The difference between Helsinki and this study might be due to the different range of temperatures in the two different geographical areas. Therefore, studies assessing the relationship between time location pattern and different temperature ranges need to be conducted over various temperature ranges.

Outdoor time location data were not determined due to the data grouped with other locations in the same category in this study, although studies in other countries have collected the time data associated with being outdoors. On average, Americans spent 7.6% of their time outdoors per day [8], while a Canadian study showed that the population spent 5.8% of their time outdoors per day, on average [11]. Due to an absence of outdoor data, outdoor time cannot be compared with other countries. Furthermore, it is not possible to estimate the time spent outdoors in the Korean population based on the personal exposure model. However, the various time location data for workplace/school and restaurant/bar are far more accurate with respect to time location patterns compared with the previous Korean studies.

## 5. Conclusions

The time activity pattern of Seoul and Busan participants was conducted in summer, fall, and winter. Time profiles and the time spent at various locations over three seasons were similar in each city. Age, gender, spouse, working status, education, monthly income, and health conditions affected the time spent at several locations. Season influenced the time spent in only two of nine locations in Seoul. Time location pattern of the Busan participants was not affected by season. The seasons affected each time location pattern for the urban populations slightly differently but overall there were few differences between the cities.

## Figures and Tables

**Table 1:** Gender and age characteristics of Seoul and Busan between study participants and original population.

Age	Seoul				Busan			
	Male		Female		Male		Female	
	Study	Population	Study	Population	Study	Population	Study	Population
10–19	281 (15.1)	479,000 (10.7)	260 (12.2)	449,179 (9.6)	134 (13.5)	168,509 (10.6)	148 (12.6)	154,088 (9.4)
20–29	270 (14.5)	718,832 (16.0)	307 (14.5)	722,809 (15.4)	104 (10.5)	239,208 (15.1)	122 (10.4)	214,338 (13.0)
30–39	328 (17.6)	810,023 (18.1)	356 (16.8)	807,109 (17.2)	155 (15.6)	245,890 (15.5)	138 (11.7)	235,290 (14.3)
40–49	337 (18.1)	832,819 (18.6)	422 (19.9)	842,082 (18.0)	177 (17.8)	276,940 (17.5)	220 (18.7)	278,973 (17.0)
50–59	293 (15.7)	768,924 (17.1)	326 (15.4)	803,014 (17.1)	191 (19.2)	295,356 (18.6)	215 (18.3)	314,094 (19.1)
60+	352 (18.9)	877,520 (19.6)	452 (21.3)	1,063,796 22.7)	233 (23.4)	361,015 (22.7)	334 (28.4)	445,765 (27.1)
Total	1861 (100)	4,487,118 (100)	2123 (100)	4,687,989 (100)	994 (100)	1,586,918 (100)	1177 (100)	1,642,548 (100)

Note: The number in bracket = (The number of participants or population in each age group)/(Total participants or population)*100.

**Table 2:** The amount of time spent by those who visited nine locations in Seoul over three seasons.

Location	Summer (*N* = 960)			Fall (*N* = 1898)			Winter (*N* = 1126)		
	Percent *	Mean ± SE **	Median ***	Percent *	Mean ± SE **	Median ***	Percent *	Mean ± SE **	Median ***
Own home	99.6%	867.7 ± 9.1	810	99.4%	843.7 ± 6.2	780	99.2%	890.0 ± 8.3	840
Workplace/school	60.1%	465.0 ± 7.7	490	62.9%	479.5 ± 5.3	490	60.0%	460.2 ± 6.9	470
Other home	5.4%	136.3 ± 22.5	90	5.7%	178.9 ± 19.0	110	6.1%	207.0 ± 26.3	120
Restaurant/bar	46.9%	85.6 ± 3.2	60	47.0%	80.5 ± 2.2	60	48.8%	83.5 ± 3.0	60
Other locations	75.1%	176.2 ± 6.3	120	73.7%	184.8 ± 4.6	140	69.8%	169.6 ± 5.9	130
Walk	77.5%	52.4 ± 1.2	50	72.2%	47.8 ± 0.9	40	69.0%	45.3 ± 1.1	40
Private transportation	24.8%	105.9 ± 5.0	90	25.1%	110.7 ± 3.5	90	29.0%	102.1 ± 4.2	80
Public transportation	45.2%	101.2 ± 2.9	90	46.3%	99.4 ± 1.9	90	40.9%	101.9 ± 2.7	90
Other transportation	6.6%	61.0 ± 4.9	50	9.5%	73.3 ± 4.3	60	7.2%	88.8 ± 10.3	60

* (The number of participants who visited this location at least for ten minutes during a day)/(Total Seoul participants)*100. ** The average time spent at this location over 24 h (1440 min) for those who visited this location. *** Median time spent at this location over 24 h (1440 min) for those who visited this location.

**Table 3:** The amount of time spent by those who visited nine locations in Busan over three seasons.

Location	Summer (*N* = 547)			Fall (*N* = 1015)			Winter (*N* = 610)		
	Percent *	Mean ± SE **	Median ***	Percent *	Mean ± SE **	Median ***	Percent *	Mean ± SE **	Median ***
Own home	100%	932.5 ± 12.3	850	99.7%	898.8 ± 8.7	860	99.8%	905.7 ± 10.7	860
Workplace/school	52.1%	461.5 ± 10.5	490	60.8%	460.1 ± 8.0	480	60.7%	451.2 ±9.9	480
Other home	3.7%	110.5 ± 19.8	75	6.2%	105.1 ± 11.8	120	8.7%	150.6 ± 21.3	120
Restaurant/bar	42.0%	74.9 ± 4.1	50	42.4%	59.9 ± 2.2	40	49.3%	58.0 ± 2.6	40
Other locations	71.7%	188.1 ± 8.8	120	79.1%	170.3 ± 4.9	140	73.9%	167 ± 7.1	140
Walk	68.2%	48.4 ± 1.7	40	69.7%	49.1 ± 1.3	40	72.3%	46.1 ± 1.5	40
Private transportation	32.7%	92.7 ± 4.0	80	24.8%	101.1 ± 4.4	80	27.7%	91.4 ± 4.5	80
Public transportation	35.8%	77.8 ± 3.1	70	38.6%	77.3 ± 2.4	60	42.5%	72.0 ± 2.5	60
Other transportation	8.2%	66.7 ± 10	50	12.6%	65.3 ± 4.5	50	11.5%	66.9 ± 5.8	50

* (The number of participants who visited this location at least for ten minutes during a day)/(Total Busan participants)*100. ** The average time amount spent at this location over 24 h (1440 min) for those who visited this location. *** Median time amount spent at this location over 24 h (1440 min) for those who visited this location.

**Table 4:** The factors that influence the time spent at six locations in Seoul.

	Own Home		Workplace/School		Restaurant/Bar		Other Locations		Private Transportation		Public Transportation	
	Adj. *R*^2^ = 0.424, *p* < 0.001		Adj. *R*^2^ = 0.448, *p* < 0.001		Adj. *R*^2^ = 0.093, *p* < 0.001		Adj. *R*^2^ = 0.057, *p* < 0.001		Adj. *R*^2^ = 0.129, *p* <0.001		Adj. *R*^2^ = 0.104, *p* < 0.001	
	*b* ± SE	*p*	*b* ± SE	*p*	*b* ± SE	*P*	*b* ± SE	*p*	*b* ± SE	*p*	*b* ± SE	*p*
Intercept	252.9 ± 29.4	<0.001	779.4 ± 28.0	<0.001	81.8 ± 5.1	<0.001	118.6 ± 19.1	<0.001	74.7 ± 6.4	<0.001	95.3 ± 6.4	<0.001
Working 1: Yes 2: No	264.1 ± 8.9	<0.001	−301.3 ± 8.4	<0.001			61.2 ± 6.6	<0.001			−33.7 ± 2.5	<0.001
Age	4.3 ± 0.2	<0.001	−4.6 ± 0.2	<0.001	−0.3 ± 0.1	<0.001	0.9 ± 0.1	<0.001	−0.1 ± 0.1	0.029		
Gender 1: Male 2: Female	84.6 ± 7.3	<0.001	−39.2 ± 6.8	<0.001	−9.2 ± 2.0	<0.001	−35.1 ± 5.5	<0.001	−12.2 ± 1.9	<0.001		
Monthly income	−9.8 ± 1.4	<0.001	7.0 ± 1.3	<0.001	3.1 ± 0.3	<0.001	−2.6 ± 1.0	0.012	3.9 ± 0.3	<0.001	−1.0 ± 0.4	0.006
Spouse 1: Yes 2: No	−39.8 ± 8.3	<0.001	39.7 ± 7.8	<0.001					−18.8 ± 2.2	<0.001	21.1 ± 2.0	<0.001
Health condition 1: Healthy 2: Poor	17.5 ± 4.2	<0.001					−10.6 ± 3.1	0.001				
Education 1: Higher than high school 2: Lower than middle school	−30.6 ± 9.1	0.001	79.8 ± 8.6	<0.001	−22.8 ± 2.5	<0.001			−10.4 ± 2.4	<0.001	−22.3 ± 2.4	<0.001
Seasons 5: Summer 6: Fall 7: Winter			10.2 ± 4.4	0.021			−10.5 ± 3.5	0.003				

**Table 5:** The factors that influence the time spent at six locations in Busan.

	Own Home		Workplace/School		Restaurant/Bar		Other Locations		Private Transportation		Public Transportation	
	Adj. *R*^2^ = 0.456, *p* < 0.001		Adj. *R*^2^ = 0.460, *p* < 0.001		Adj. *R*^2^ = 0.095, *p* < 0.001		Adj. *R*^2^ = 0.084, *p* < 0.001		Adj. *R*^2^ = 0.257, *p* < 0.001		Adj. *R*^2^ = 0.076, *p* < 0.001	
	*b* ± SE	*p*	*b* ± SE	*p*	*b* ± SE	*p*	*b* ± SE	*p*	*b* ± SE	*p*	*b* ± SE	*p*
Intercept	305.1 ± 36.6	<0.001	436.6 ± 78.0	<0.001	59.6 ± 5	<0.001	536.0 ± 54.0	<0.001	74.2 ± 7.7	<0.001	53.8 ± 6.2	<0.001
Working 1: Yes 2: No	233.9 ± 11.8	<0.001	−277.9 ± 11.4	<0.001			72.1 ± 6.4	<0.001	−9.3 ± 2.6	<0.001	−10.1 ± 2.5	<0.001
Age	6.0 ± 0.3	<0.001	−5.6 ± 0.3	<0.001	−0.3 ± 0.1	<0.001			−0.3 ± 0.1	<0.001		
Gender 1: Male 2: Female	70.1 ± 9.6	<0.001	10.5 ± 2.0	<0.001	−11.4 ± 2.0	<0.001	−35.1 ± 6.4	<0.001	−13.8 ± 2.2	<0.001		
Monthly income	−12.4 ± 2.2	<0.001			2.6 ± 0.4	<0.001			6.0 ± 0.5	<0.001	−2.2 ± 0.4	<0.001
Spouse 1: Yes 2: No	−50.6 ± 10.4	<0.001	41.7 ± 10.0	<0.001					−15.3 ± 2.3	<0.001	17.5 ± 2.1	<0.001
Health condition 1: Healthy 2: Poor	11.9 ± 5.8	0.04										
Education 1: Higher than high school 2: Lower than middle school	−29.6 ± 11.5	0.01	46.8 ± 11.1	<0.001	−9.3 ± 2.3	<0.001					−20.3 ± 2.2	<0.001
Seasons 5: Summer 6: Fall 7: Winter												
Agricultural 1: Yes 2: No			172.2 ± 36.6	<0.001			−230.5 ± 26.6	<0.001				

## Supplementary Materials

The following are available online at www.mdpi.com/1660-4601/14/7/672/s1, Table S1: The factors that influence the time spent at other 3 locations in Seoul. Table S2: The factors that influence the time spent at other 3 locations in Busan.
