# Supplementary material for: Seasonal Differences in Determinants of Time Location Patterns in an Urban Population: A Large Population-Based Study in Korea
Source: Int J Environ Res Public Health. 2017 Jun 22;14(7):672. doi: 10.3390/ijerph14070672 (PMC5551110; doi:10.3390/ijerph14070672)
Supplement: Supplementary file 1 [file ijerph-14-00672-s001.pdf]

S1. The factors that influence the time spent at other 3 locations in Seoul.

	**others' home**		**walk**		**other transportation**	
	Adj. R^2= 0.014 P<0.001		Adj. R^2 = 0.045 P<0.001		Adj. R^2= 0.018 P<0.001	
	b±SE	P	b±SE	P	b±SE	P
**Intercept**	-29.6±5.3	<0.001	46.1±3.6	<0.001	13.2±2.0	<0.001
**Working**						
1 : Yes	7.2±2.0	<0.001				
2: No						
**Age**	0.2±0.1	<0.001	-0.2±0.0	<0.001	-0.1±0.0	<0.001
**Gender**						
1: Male	9.2±2.0	<0.001	4.9±1.2	<0.001	-5.8±0.9	<0.001
2: Female						
**Monthly income**			-0.7±0.2	<0.001		
**Spouse**						
1: Yes						
2: No						
**Health condition**						
1 : Healthy			-2.8±0.7	<0.001		
2 : Poor						
**Education**						
1 : Higher than high school			6.7±1.4	<0.001	5.1±1.1	<0.001
2 : Lower than middle school						
**Seasons**						
5: Summer<br>6: Fall<br>7 : Winter	2.7±1.4	0.049	-4.2±0.7	<0.001		

S2. The factors that influence the time spent at other 3 locations in Busan.

	**others' home**		**walk**		**other transportation**	
	Adj. R^2= 0.014 P<0.001		Adj. R^2 = 0.046 P<0.001		Adj. R^2= 0.036 P<0.001	
	b±SE	P	b±SE	P	b±SE	P
**Intercept**	-20.8±4.4	<0.001	34.7±4.7	<0.001	67.3±10.4	<0.001
**Working**						
1 : Yes					-4.4±1.3	<0.001
2: No						
**Age**			-0.2±1.0	<0.001	-0.2±0.0	<0.001
**Gender**						
1: Male	4.9±1.8	0.007			-5.4±1.2	<0.001
2: Female						
**Monthly income**			-1.0±0.3	<0.001		
**Spouse**						
1: Yes			4.8±1.7	0.005		
2: No						
**Health condition**						
1 : Healthy			-2.5±1.9	0.005		
2 : Poor						
**Education**						
1 : Higher than high school	9.0±2.0	<0.001	8.0±1.9	<0.001	6.5±1.4	<0.001
2 : Lower than middle school						
**Seasons**						
5: Summer<br>6: Fall<br>7 : Winter	4.6±1.2	<0.001				

**Agricultural**		
1: Yes	-23.2±5.0	<0.001
2. No		
